# The clinical relevance of heme detoxification by the macrophage heme oxygenase system

**DOI:** 10.3389/fimmu.2024.1379967

**Published:** 2024-03-22

**Authors:** Scott Yeudall, Clint M. Upchurch, Norbert Leitinger

**Affiliations:** ^1^ Department of Pharmacology, University of Virginia School of Medicine, Charlottesville, VA, United States; ^2^ Medical Scientist Training Program, University of Virginia School of Medicine, Charlottesville, VA, United States; ^3^ Department of Neuroscience, Center for Brain Immunology and Glia (BIG), University of Virginia School of Medicine, Charlottesville, VA, United States; ^4^ Robert M Berne Cardiovascular Research Center, University of Virginia School of Medicine, Charlottesville, VA, United States

**Keywords:** heme, oxidative stress, inflammation, ferroptosis, cardiometabolic disease, hemoglobin, iron, hemolysis

## Abstract

Heme degradation by the heme oxygenase (HMOX) family of enzymes is critical for maintaining homeostasis and limiting heme-induced tissue damage. Macrophages express HMOX1 and 2 and are critical sites of heme degradation in healthy and diseased states. Here we review the functions of the macrophage heme oxygenase system and its clinical relevance in discrete groups of pathologies where heme has been demonstrated to play a driving role. HMOX1 function in macrophages is essential for limiting oxidative tissue damage in both acute and chronic hemolytic disorders. By degrading pro-inflammatory heme and releasing anti-inflammatory molecules such as carbon monoxide, HMOX1 fine-tunes the acute inflammatory response with consequences for disorders of hyperinflammation such as sepsis. We then discuss divergent beneficial and pathological roles for HMOX1 in disorders such as atherosclerosis and metabolic syndrome, where activation of the HMOX system sits at the crossroads of chronic low-grade inflammation and oxidative stress. Finally, we highlight the emerging role for HMOX1 in regulating macrophage cell death via the iron- and oxidation-dependent form of cell death, ferroptosis. In summary, the importance of heme clearance by macrophages is an active area of investigation with relevance for therapeutic intervention in a diverse array of human diseases.

## Macrophages: master regulators of systemic heme homeostasis

Heme is widely distributed throughout the cell as an essential component of globins (including hemoglobin, myoglobin, and neuroglobin), numerous enzymes (including oxidases, catalases, and reductases), and electron carriers such as cytochrome proteins (1). In all these cases the iron coordinated in the porphyrin core of the heme molecule allows for oxygen binding and/or electron transfer via oxidation-reduction reactions that facilitates the function of these heme-containing proteins. The redox reactivity of heme is a double-edged sword, and when present in high enough amounts leads to cellular damage via oxidation of intracellular proteins, lipids, and other molecules. As such, the concentration and localization of heme in cells is tightly controlled. Accumulation of heme is tightly regulated though binding to their associated proteins such as hemoglobin, but heme moieties can be released upon oxidation of the iron complexed within the heme molecule, generating pro-oxidant iron in the Fe3+ or Fe4+ state (2). The critical role for oxidation in releasing free heme from hemoglobin has been demonstrated *in vitro* as well as cell culture conditions, where it has been demonstrated that exposure of endothelial cells to previously oxidized hemoglobin induces a greater level of oxidative damage compared with fully reduced hemoglobin (3).

**Figure 1:**
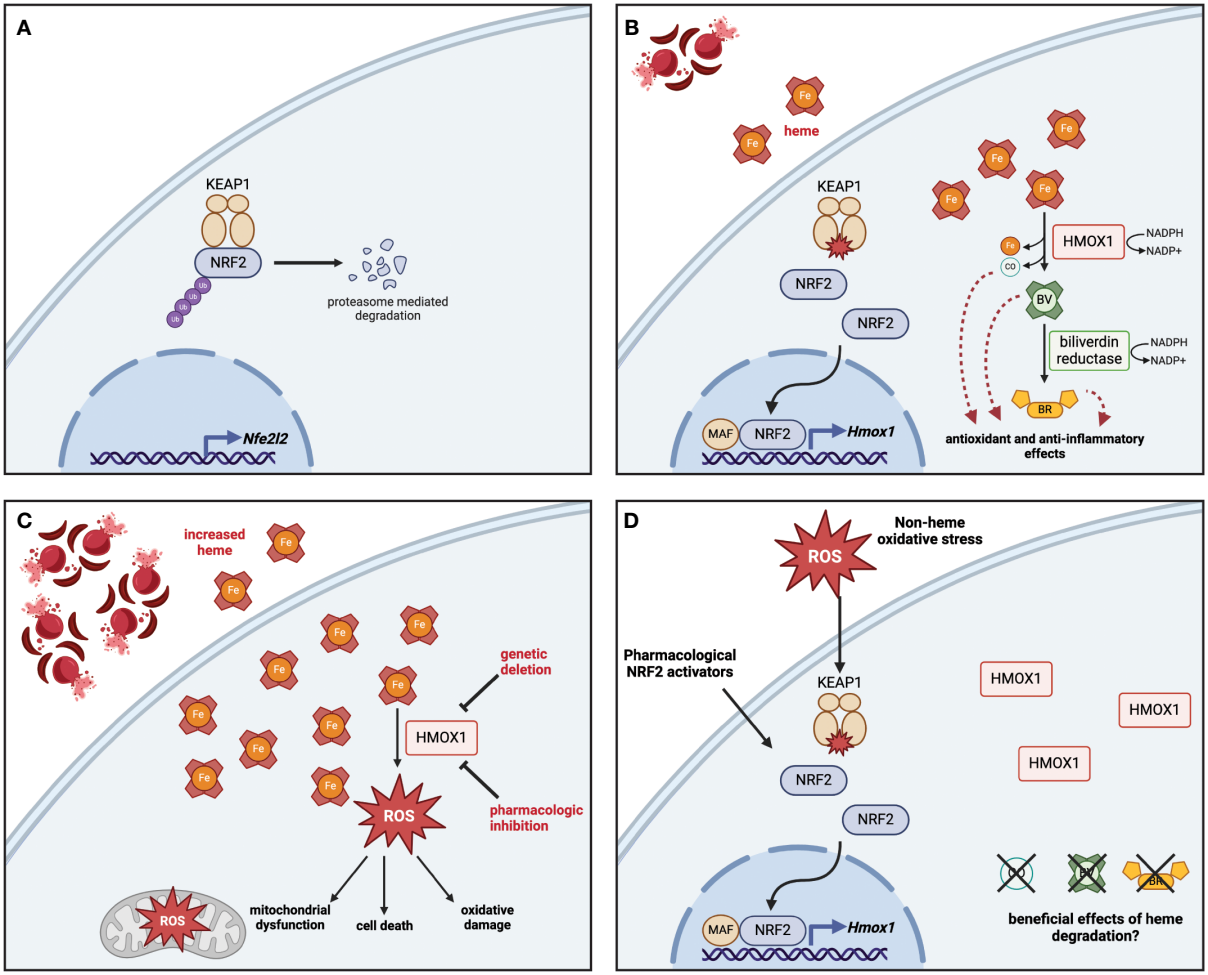
Heme detoxification via NRF2-dependent HMOX1 protects against oxidative stress and releases byproducts with beneficial effects. **(A)** At rest, NRF2 is sequestered in the cytoplasm bound to KEAP1, which polyubiquitinates NRF2 to promote its proteasomal degradation. The base level of NRF2 is maintained by low-level expression of the *Nfe2l2* gene. **(B)** In response to heme, the oxidation of key cysteine residues on KEAP1 release NRF2, which travels to the nucleus and interacts with small MAF proteins to promote the transcription of antioxidant genes including *HMOX1*. The enzymatic activity of HMOX1 converts heme into biliverdin, releasing iron and carbon monoxide (CO) as byproducts; biliverdin is then converted to bilirubin by biliverdin reductase. Both enzymes depend on the availability of NADPH as a cofactor. **(C)** Genetic deletion or pharmacologic inhibition of HMOX1 activity results in heme accumulation, which leads to increased reactive oxygen species (ROS) production, driving mitochondrial dysfunction, oxidative damage, and in some cases cell death. **(D)** Although non-heme oxidative stress or pharmacologic activation of NRF2 can lead to HMOX1 upregulation, the beneficial effects of heme degradation require heme to be broken down into CO, biliverdin, and bilirubin. Figure created with BioRender (http://biorender.com).

The heme oxygenase system is critical for the regulation of the intracellular heme pool, degrading heme into its breakdown product biliverdin and in the process releasing iron, which can be sequestered via binding to ferritin, and carbon monoxide (CO), which acts as an intracellular signaling molecule (4, 5). Two isoforms of the heme oxygenase enzyme are found in mammals, which share a common catalytic mechanism requiring the cofactor NADPH. Heme oxygenase 2 (HMOX2) is constitutively expressed in a majority of cell types throughout the body to provide a basal level of heme degradation needed to regulate the local intracellular heme pool. Conversely, heme oxygenase 1 (HMOX1) is induced in response to increased intracellular levels of heme or other oxidative stresses, primarily via the activation of the redox-sensitive transcription factor nuclear factor erythroid 2-related factor 2 (NRF2), although other transcription factors have been shown to play a role in its expression in a variety of cell types (6–11). This two-isoform system allows for both maintenance of intracellular heme homeostasis throughout the organism as well as an increased capacity for heme degradation on-demand in response to higher levels of heme, either acutely or chronically (The activation of the HMOX system in response to heme via NRF2 is summarized in **Figure 1**).

In addition to activation by NRF2, the expression of HMOX1 is controlled by the transcriptional repressor BTB and CNC homology 1 (BACH1), which under normal conditions binds as a heterodimer with MafK to enhancer regions of the *HMOX1* locus at Maf recognition elements (MAREs), where it acts to limit expression when heme levels are low (12). Heme present in the cell can directly bind to BACH1, resulting in detachment of BACH1 from DNA and nuclear export (13), where it is then polyubiquitinated and degraded by the proteasome (14). Knockout of *Bach1* in mice results in increased basal expression of HMOX1 (15), demonstrating that heme-dependent control of BACH1 activity is sufficient to activate the heme detoxification system. Although most work has focused on the role of BACH1 in control of HMOX1 expression, more recent work has demonstrated that it can also act as a repressor of other redox-responsive genes, particularly those involved in glutathione synthesis (16). Together, this demonstrates the nuanced role of multiple transcription factors in regulating *HMOX1* expression.

Macrophages play a central role in heme oxygenase-mediated maintenance of systemic heme homeostasis. Macrophages of the reticuloendothelial system are responsible for the phagocytic uptake and degradation of aged or damaged erythrocytes, which contain large amounts of heme bound to hemoglobin (17). This process, known as erythrophagocytosis, is largely performed by red pulp macrophages in the spleen, which are characterized by a transcriptional program notable for high levels HMOX1 expression as well as the cell-surface hemoglobin-haptoglobin scavenger CD163 (18). Heme drives the development and maintenance of red pulp macrophages through inducing the expression of the transcription factor Spi-C, which is required both for initial development of red pulp macrophages from the embryologic yolk sac, as well as differentiation of monocytes into iron-recycling macrophages that can replenish the red pulp macrophage population during pathologic increases in systemic heme levels (19, 20). The liver is also a site of erythrocyte clearance, particularly in response to acute hemolysis (21), where liver-resident Kupffer cells are supported by monocyte-derived macrophages expressing high levels of ferroportin to facilitate iron transfer to hepatocytes (22).

The critical importance of macrophages in maintenance of heme and iron homeostasis has been demonstrated through a combination of mouse-based genetic approaches and analysis of the consequences of human *HMOX1* mutations. Global deletion of *Hmox1* in mice leads to a phenotype characterized by iron overload, chronic inflammation, and increased markers of oxidative stress (23). Notably, mice lacking HMOX1 globally had significantly reduced numbers of splenic and hepatic macrophages, which was hypothesized to be a result of chronic heme exposure leading to heme-induced death in these cells (24). Further studies demonstrated the essential role of myeloid HMOX1 in systemic protection against heme-induced damage, as transplantation of wild-type bone marrow or infusion of wild-type macrophages rescued both reticuloendothelial macrophage populations as well as reduced inflammation and iron overload in *Hmox1^-/-^* mice (25, 26). In addition, multiple cases of human HMOX1 deficiency have been reported with pathological findings including chronic hemolytic anemia, developmental delay, asplenia, renal tubular injury, and early mortality (27–30). In one case, low-density lipoprotein (LDL) isolated from a patient with HMOX1 deficiency induced cytotoxicity in endothelial cells *in vitro*, supporting a role for increased free heme in driving oxidation of LDL and furthering inflammatory and oxidative damage (31). These observations underscore the importance of the heme oxygenase system in maintaining whole-body redox homeostasis and highlight macrophages as central regulators of heme degradation by HMOX1.

## Macrophage heme oxygenase in hemolytic disorders

Hemolysis, whether acute or chronic, places increased demand on the heme oxygenase system, as massive amounts of hemoglobin are released from damaged erythrocytes into the circulation. Efficient degradation of heme by macrophages is critical to prevent oxidative tissue damage, which can lead to chronic inflammation and exacerbate the underlying disorders. As such, a significant amount of work has been done to elucidate the critical role of the heme oxygenase system in acute and chronic hemolytic disorders such as sickle cell disease, thalassemias, enzymopathies including pyruvate kinase and glucose-6-phosphate dehydrogenase (G6PD) deficiency, autoimmune hemolytic anemias, and trauma-induced hemolysis.

Sickle Cell Disease (SCD) is an inherited hemoglobinopathy where a point mutation in the beta-globin gene produces a defective form of hemoglobin that form insoluble aggregates when deoxygenated, which results in the prototypical “sickle” shape of affected erythrocytes and significantly decreases erythrocyte longevity (32). Sickle erythrocytes are prone to hemolysis and consequently patients with SCD suffer oxidative tissue damage from chronically high heme levels, as well as vaso-occlusive exacerbations that lead to acute inflammation, tissue infarction, and ultimately organ fibrosis (33). Work in animal models of SCD have demonstrated that extracellular heme is sufficient to drive vaso-occlusion, intravascular hemolysis, and lung pathology that mimics acute chest syndrome (34–36). Chronic inflammation driven by heme leads to an overall pro-inflammatory polarization of macrophages in patients with SCD, as well as SCD mouse models (37). At the same time, both tissue macrophages and circulating monocytes from SCD patients have higher levels of HMOX1 compared to control subjects (38, 39). HMOX1-expressing monocytes have been shown to scavenge damaged erythrocytes from the endothelium, and an increase in this specific population in SCD patients is associated with a decreased rate of vaso-occlusive crisis (38). However, the polarization of monocytes by heme not only promotes an antioxidant patrolling phenotype via NRF2, but can also promote differentiation of monocytes into proinflammatory monocyte-derived macrophages via type I interferon-dependent upregulation of Chemokine (C-C motif) Ligand-2 (CCL2) (40). These activated monocyte-derived macrophages are recruited to the liver, where they upregulate Fc receptors and have increased capacity for antibody-mediated erythrophagocytosis (41).

In addition to circulating monocytes responding to hemolysis within the vasculature, tissue-resident macrophages have also been shown to drive pathology through maladaptive responses to heme. A hemoglobin-polarized population of perivascular macrophages with a mixed antioxidant/vasoactive/inflammatory phenotype and significant intracellular iron accumulation was identified in the lungs of SCD patients with concomitant pulmonary hypertension (PH), and a similar population of macrophages was identified in a rat model of PH induced by a combination of free hemoglobin administration and hypoxic stress (39). Tissue macrophages in mouse models of SCD also have an impaired ability to engulf and clear apoptotic cells, which is driven by heme-induced suppression of PPAR-gamma-PGC1-alpha signaling and a reduced capacity to switch their metabolism to beta oxidation (42). Moreover, reactivation of these metabolic pathways improved efferocytotic capacity, which could serve as a therapeutic strategy to promote resolution of inflammation in SCD (42). Additionally, macrophages in the liver of SCD mice have a proinflammatory transcriptional profile that is driven in part by reactive oxygen species formation, and administration of exogenous hemopexin reduces expression of inflammatory markers (37). Indeed, multiple studies have demonstrated the ability of hemopexin or haptoglobin treatment to reduce inflammation and tissue damage in preclinical models of SCD (34, 37, 43, 44).

The breakdown of heme via heme oxygenase not only sequesters pro-oxidant iron and reduces the pro-inflammatory impact of free heme, but also releases biliverdin and carbon monoxide (CO), molecules which have been shown to have significant antioxidant and anti-inflammatory effects (4, 5). As such, the use of either NRF2 activators to promote HMOX1 activity or direct administration of CO have been employed to treat inflammation and promote redox homeostasis in SCD (45, 46). Administration of inhaled CO reduces leukocytosis, attenuated inflammation and decreases vaso-occlusion in SCD mice (47, 48), while daily oral administration of a saturated liquid formulation of CO enhanced NRF2 and HMOX1 expression and decreased NF-κB activation (49). This oral form of CO also increased hemoglobin levels and protected against hypoxia reoxygenation-induced vaso-occlusion (49). In addition, strategies that take advantage of the ability of hemoglobin to bind CO have been used for therapeutic delivery of CO in SCD mice, with similar activation of HMOX1, decreased inflammatory signaling, and protection against vaso-occlusion (50). These benefits could be blocked by administration of the HMOX1 inhibitor tin protoporphyrin, underlying the importance of HMOX1 activity for the beneficial effects of CO in this model. These studies have highlighted the importance of continuous heme oxygenase activity in both breaking down the elevated levels of heme as well as promoting redox homeostasis and reducing chronic inflammation in sickle cell disease.

The importance of heme clearance by macrophages has also been examined in a variety of other hemolytic disorders, including inherited metabolic enzymopathies, erythrocyte structural defects, and acquired hemolytic diseases. Deficiency of glucose-6-phosphate dehydrogenase (G6PD), which catalyzes the first step of the pentose phosphate pathway (PPP), is the most common inherited enzyme defect worldwide (51). In response to infection, inflammation, or increased oxidative stress from certain medications, patients with G6PD deficiency experience acute intravascular hemolysis (51), a finding that has also been recapitulated in mouse models of the disorder (52–55). As the first step of the oxidative branch of the PPP, the enzymatic activity of G6PD is a major source of NADPH within cells, and therefore is critical for maintenance of redox homeostasis (56). This is particularly important in erythrocytes, which are under a constant high level of oxidative stress. In macrophages, exposure to heme induces the PPP at the transcriptional and functional level, which provides the NADPH needed to fuel HMOX1 activity; as a result, inhibition of G6PD impairs heme degradation (57). The importance of PPP activity for heme clearance by macrophages suggests a multifaceted impact of G6PD deficiency – not only are patients at increased risk of hemolysis, but they may also be unable to degrade the free heme effectively. Increased free heme has also been shown to polarize myeloid cells toward heme-clearing phenotypes: In a genetic model of hereditary spherocytosis, chronic heme stress shifted the transcriptional profile of dendritic cells toward that of splenic red pulp macrophages through activation of NRF2 signaling (58), while in the same model liver macrophages had an anti-inflammatory, erythrophagocytic phenotype (21).

## Heme degradation modulates infection, sepsis, and hyperinflammation

Heme sensing by cells has been demonstrated to activate inflammatory intracellular signaling pathways. Heme directly agonizes Toll-like receptor (TLR) 4 in macrophages, which leads to activation of the NF-κB signaling cascade, increased inflammatory gene expression, and release of cytokines including TNFα (59). This work also suggested that heme does not directly compete for binding with lipopolysaccharide (LPS), a canonical TLR4 ligand, and further studies have identified heme binding sites on the myeloid differentiation factor 2 (MD-2) protein, which facilitate the activation of TLR4 signaling (60, 61). The clinical relevance of heme-induced TLR4 activation has been demonstrated in murine SCD, where genetic deletion or pharmacological inhibition of TLR4 prevented heme-induced vaso-occlusion, mainly through activation of endothelial cells (35). Moreover, in an *ex vivo* model of thrombo-inflammation in human blood, inhibition of the TLR4 coreceptor CD14 attenuated heme-induced complement activation and cytokine release (62). These and multiple other studies have demonstrated the role of TLR activation in heme-driven inflammation in both mice and humans (63).

In addition to activation of inflammatory gene expression via TLR-NF-κB signaling, heme can also trigger cytokine release via activation of caspase-dependent inflammasomes (64). Heme has been shown to induce the processing of interleukin-1β (IL-1β) by caspase 1 in LPS-primed macrophages by the NLRP3 inflammasome via a mechanism that is dependent on intracellular ROS production and spleen tyrosine kinase (65). As a result, mice with genetic deletion of key inflammasome components were protected from lethality in a model of chemically-induced sterile hemolysis. In addition, heme can induce activation of caspase-4 and caspase-5, leading to IL-1β release, a process that was independent of the canonical inflammasomes (66). Furthermore, recent work demonstrated a role for heme in driving a novel form of programmed inflammatory cell death coined panoptosis, which was dependent on the NLRP12 inflammasome (67). These data support a role for heme modulating the response to other pro-inflammatory signals, with implications in a broad range on inflammatory pathologies.

The role of heme as a driver of oxidative tissue damage and inflammation and the critical importance of heme clearance has also been demonstrated in models of acute bacterial infection. *Hmox1* knockout mice succumbed to a low-grade polymicrobial infection that was not lethal in wild-type mice (68). Infection increased circulating heme levels, and administration of heme exacerbated damage in severe sepsis independent of bacterial burden, and an analysis of patients who died of septic shock also showed increased circulating heme levels (68). The activity of heme oxygenase in macrophages is also critical for controlling infection in sepsis, as mice with a myeloid-specific deletion of *Hmox1* had higher bacterial burden and decreased survival in a model of *E. coli* peritonitis (69). Mechanistically, heme impairs bacterial engulfment by macrophages through disruption of cytoskeletal rearrangement required for phagocytosis, and blocking this disruption attenuated heme-induced pathology in this model. In a combined trauma-infection model, increased circulating heme induced by traumatic liver crush injury impaired clearance of *Staphylococcus aureus* bacteria from the lung by polymorphonuclear leukocytes (70).

Moreover, clearance of heme is critical to control bacterial infection since free heme is taken up by bacteria as a source of iron, which is critical for bacterial growth and division (71). Multiple pathogenic bacterial species including *S. aureus*, *E. coli*, and *Streptococcus pyogenes* have heme-binding and/or heme-uptake proteins that have been shown to contribute to virulence (72–74). Accordingly, heme oxygenase activity in macrophages is critical for control of intracellular infection with *Mycobacterium*, as *Hmox1* deficient mice have higher pathogen burdens and fail to mount a granulomatous response to control *M. avium*. *Hmox1* deficient mice die at a low dose of *M. tuberculosis* that is tolerated in mice which express *Hmox1*, through a mechanism that seemed to be driven by heme-induced cellular death of infected macrophages (75). Conversely, *M. tuberculosis* infection has been shown to induce HMOX1 expression in mouse and human macrophages, and chemical inhibition of HMOX1 restricted mycobacterial growth and cytokine production (76). This detrimental role for HMOX1 activity in either promoting pathogen replication or impairing the immune response to infection has also been seen in several other bacterial and protozoan pathogens, including *Salmonella typhimurium* (77), *Brucella* (78), *Burkholderia pseuodmallei* (79), and *Leishmania chagasi* (80, 81). In many of these cases, the activity of HMOX1 and downstream products including CO suppress production of reactive oxygen species that are needed for the oxidative respiratory burst and augment the activation of pattern recognition receptor-dependent innate immune signaling. These studies highlight that the context and timing of heme degradation by heme oxygenase are critical for determining whether this process positively or negatively impacts the host response to infection.

Although heme promotes inflammation either via direct activation of innate immune signaling pathways or impairing phagocytic functions, there is also growing evidence that, in certain contexts, heme can have anti-inflammatory effects, mainly as a result of heme-dependent activation of the heme oxygenase system, particularly increased HMOX1 activity (82). *Hmox1* deficient mice had increased levels of pro-inflammatory cytokines and a hyperinflammatory response to LPS exposure (83), and conditional deletion of *Hmox1* in myeloid cells resulted in an exacerbated inflammatory phenotype in the experimental autoimmune encephalomyelitis (EAE) model of multiple sclerosis, which is dependent on activation of interferon beta signaling (84). In mouse alveolar macrophages, heme treatment induced an anti-inflammatory phenotype in the context of acute lung injury, through a mechanism involving increased phagocytic clearance and decreased iNOS activity, and this could be blocked by treatment with zinc protoporphyrin, suggesting that HMOX1 activity was critical for this effect (85). Further evidence that the anti-inflammatory effect of heme depends on enzymatic breakdown by HMOX1 has come from studies demonstrating that heme breakdown products, namely carbon monoxide and biliverdin/bilirubin, have anti-inflammatory effects (86–88). Indeed, a patient with a mutant form of HMOX1 that resulted in decreased biliverdin (and therefore) bilirubin production, had a hyperinflammatory phenotype resembling hemophagocytic lymphohistiocytosis (89). Furthermore, uptake of hemoglobin-haptoglobin complexes by CD163 resulted in polarization of macrophages toward an anti-inflammatory phenotype with a distinct antioxidant component (90). These and other studies have demonstrated the critical requirement for HMOX1 enzymatic activity in mediating the anti-inflammatory effects of heme under specific conditions.

The COVID-19 pandemic, caused by infection with the novel coronavirus SARS-CoV-2, has reignited interest in understanding how dysregulation of the immune response can impact the clinical course of acute viral infections. In a single-center study of patients with COVID-19, higher circulating heme and heme oxygenase protein levels were observed in patients who developed sepsis (91), and an increase heme levels over time was also observed in patients with more severe oxygen desaturation (92). Interestingly, in a recent multi-center, longitudinal study of patients who went on to develop persistent symptoms months after acute COVID-19, termed “long COVID”, circulating heme levels were increased, raising the intriguing possibility that heme-driven inflammation might be one of a multitude of factors contributing to chronic thrombo-inflammation in this disease (93). Continuing work in this area may identify a mechanistic role for heme in driving the severity of acute infection or persistence of inflammation that could provide opportunities for therapeutic intervention. In summary, heme clearance by macrophages modulates hyperinflammation and the response to infection, with implications for sterile inflammatory insults, bacterial, parasitic, and viral infections.

## Metaflammation and cardiometabolic disease

Beyond hemolytic disorders and acute inflammatory insults, the heme oxygenase system has been implicated in development and progression of chronic cardiometabolic diseases, including atherosclerosis and obesity-induced insulin resistance, diseases in which macrophages have been shown to play a central pathological role. Macrophages in the core of the atherosclerotic plaque are responsible for the uptake and clearance of pro-inflammatory materials including oxidized LDL and associated lipid oxidation products, oxidatively-modified proteins, and damaged erythrocytes. Free heme is sufficient to oxidize LDL (oxLDL), which contributes to endothelial injury during the pathogenesis of atheroma development (94). Minimally-modified LDL was shown to induce HMOX1 expression in both endothelial cells and macrophages, and augmenting HMOX1 expression with heme attenuated oxLDL-induced monocyte chemotaxis (95). Once in the lesion, macrophage heme oxygenase activity modulates the progression of inflammation and the maintenance of lesion stability. Particularly in the necrotic core of the lesion, where microscopic hemorrhage is associated with decreased plaque stability and increased likelihood of rupture (96, 97). The interaction of accumulated lipids with erythrocytes in regions of microhemorrhage in the core of the lesion also drives hemolysis and oxidation of heme iron which facilitates liberation of heme and contributes to overall oxidative stress in atherosclerotic lesions (98).

Release of heme from intraplaque hemorrhage induces an atheroprotective macrophage phenotype, which is dependent on a positive feedback loop driven by interleukin-10 (99). This adaptive macrophage phenotypic polarization is driven by heme-dependent activation of NRF2, which results in decreased markers of oxidative stress (100). Moreover, heme activates ATF1 signaling to induce HMOX1 expression, and activation of this signaling pathway in intraplaque macrophages attenuates foam cell formation (9, 101). A similar pathway is also critical for hematoma clearance by macrophages, which has implications for traumatic injury as well as microscopic hemorrhage (102). On the other hand, oxidized ferri-hemoglobin in atherosclerotic lesions has been shown to drive pro-inflammatory and pro-atherogenic polarization of macrophages (103). Oxidized hemoglobin also inhibits the upregulation of genes associated with an osteoclast-like macrophage phenotype in the necrotic core, which may modulate plaque calcification (104, 105). In addition, macrophages polarized by exposure to oxidized phospholipids upregulate HMOX1 and other key antioxidant enzymes via an NRF2-dependent process, and these redox-responsive macrophages are highly abundant in advanced atherosclerotic lesions in *Ldlr*-deficient mice (106). As a result, genetic deletion of HMOX1 exacerbates atherosclerosis in the *Ldlr-*knockout model (107), and the critical role for macrophages in this process was further demonstrated using myeloid-ablation/reconstitution experiments with bone marrow from control or *Hmox1*-knockout mice (108). The importance of HMOX1 expression in cardiovascular disease extends to human patients, as polymorphisms in the *HMOX1* promotor correlate with clinical outcomes in cardiovascular pathologies including abdominal aortic aneurysm formation (109), cerebrovascular ischemia (110), and restenosis after balloon angioplasty (111–114).

Macrophages also control the inflammatory state of the adipose tissue in obesity, which in turn impacts local tissue and systemic insulin resistance and development of other metabolic syndrome-associated disorders including hypertension, diabetes, and metabolic-associated steatotic liver disease (MASLD) (115–118). HMOX1-expressing macrophages comprise a large fraction of the tissue-resident macrophages in white adipose tissue in lean mice, and they have a bioenergetic and transcriptional profile that is dominated by activation of antioxidant pathways (119). In response to high-fat feeding, these HMOX1-expressing macrophages remain in the adipose tissue, but are outnumbered by infiltrating monocyte-derived macrophages which have increased reliance on glycolytic metabolism and a proinflammatory phenotype. Induction of HMOX1 expression in adipose macrophages by hemin treatment decreased adipose tissue inflammation in a high-fat diet model, and this protective effect could be blocked by inhibition of HMOX1 (120). Conversely, irradiated mice that were reconstituted with HMOX1-haploinsufficient bone marrow had decreased macrophage infiltration into the adipose tissue in response to high-fat feeding, resulting in improved peripheral insulin sensitivity, revealing a nuanced role for HMOX1 in control of inflammation in obese adipose tissue (121). In spontaneously hypertensive rats, induction of HMOX1 activity by hemin reduced inflammation and improved insulin sensitivity via polarization of macrophages toward a more anti-inflammatory state, an effect that was blocked by inhibition of HMOX1 by chromium-mesoporphyrin (122). Conversely, genetic deletion of HMOX1 in macrophages or hepatocytes protected mice from development of insulin resistance and inflammation in a model of diet-induced obesity (123). Importantly, this study also demonstrated that increased HMOX1 expression was predictive of insulin resistance in a cohort of obese patients (123), suggesting that the presence of HMOX1 expression is not necessarily indicative of a protective antioxidant state, and is context- and cell type-specific. These studies demonstrate the importance for HMOX1 activity in modulating the inflammatory state of macrophages in adipose tissue, which in turn has identified novel therapeutic strategies to reduce visceral inflammation in diet-induced metabolic syndrome.

## Beyond heme clearance: HMOX1 and regulation of ferroptosis and iron-induced damage

The canonical role of HMOX1 is to convert free heme to biliverdin, resulting in the release of carbon monoxide and iron, which have disparate effects on the cell. On the one hand, CO is viewed as anti-inflammatory and contributes to “silent” resolution of oxidative and inflammatory damage that results from elevated levels of free heme (4, 124). On the other hand, free iron can contribute to oxidative damage if not sequestered by ferritin. As a result, the protective effect of heme breakdown by HMOX can prove detrimental in instances where secondary protective mechanisms, which are required to minimize oxidative damage from the released, are compromised (125–127). In these cases, HMOX1-dependent release of iron may serve as pro-oxidant stimulus and drive the oxidative form of cell death, ferroptosis. Emerging literature supports the idea of a Janus-faced role for HMOX1 in regulating ferroptosis, with evidence that it not only can protect from oxidative cell death, but also induce ferroptosis in specific contexts, including endotoxin-induced inflammation, sterile injury, and metabolic syndrome.

In models of inflammation, activity of HMOX1 has been shown to both promote and attenuate pathological ferroptosis depending on the context. *Hmox1* expression is increased in microglia in response to systemic LPS treatment, and this is exacerbated in aged mice (128). Myeloid-specific deletion of *Hmox1* resulted in restored iron metabolism and reduced markers of oxidative stress in microglia from aged mice challenged with LPS. As a result, myeloid-specific *Hmox1* knockout mice were protected from neurobehavioral deficits induced by systemic inflammation (128). Conversely, in a model of LPS-induced acute lung injury, pharmacological suppression of the ferroptosis-promoting enzyme *Acsl4* protected against injury; in this model the expression of canonical ferroptosis genes *Gpx4* and *Slc7a11* as well as *Hmox1* were increased (129). In a cohort of patients with *M. tuberculosis* infection, HMOX1 levels were increased (130). *In vitro*, macrophages infected with *Mycobacterium* exhibited significantly upregulated *Hmox1* levels, coincident with an increase in intracellular iron and lipid peroxide levels. However, siRNA-mediated knockdown of *Hmox1* during infection increased bacterial release and ferroptotic cell death. These conflicting findings may be accounted for by the role of iron metabolism in replication of *M. tuberculosis in vivo* (130).

Heme oxygenase-dependent ferroptosis also drives pathology in multiple sterile injury models. *Hmox1* expression is regulated by FoxO3a-mediated autophagy *in vitro* in BV2 microglia, and in response to hemin treatment microglia upregulated *Hmox1*, which resulted in iron accumulation, lipid peroxidation, and ferroptosis (131). Virus-mediated knockdown of *FoxO3a* in the brain resulted in decreased expression of *Hmox1* in the striatum and cortex of mice in a model of intracerebral hemorrhage. Concomitantly, these mice also exhibited improved recovery post-hemorrhage (131). *In vitro*, alternatively-activated macrophages were more susceptible to cigarette smoke extract-induced ferroptotic cell death compared to classically activated macrophages. Inhibition of HMOX1 using zinc protoporphyrin protected alternatively activated macrophages from ferroptotic cell death suggesting that oxidative injury may drive ferroptotic injury in response to smoke inhalation (132). In a mouse model of endometriosis, which is characterized by chronic sterile inflammation and oxidative stress, levels of HMOX1 in peritoneal macrophages were upregulated and correlated with an increase iron content in endometriosis tissue (133). Additionally, THP-1 cells, a human monocytic cell line polarized *in vitro* by stimulation with PMA and cyst fluid from mice with endometriosis were more likely to die by ferroptosis. This suggests a correlative role between *Hmox1* levels and macrophage ferroptosis in this disease (133).

The role for ferroptosis in modulating macrophage function in cardiovascular disease is less clear. In one study, *Hmox1* expression was reduced in macrophage foam cells leading to increased oxidative stress (134). In another study, metformin treatment lowered macrophage HMOX1 expression *in vitro* after co-stimulation with oxLDL. Concomitantly, metformin-treated macrophages had lower levels of malondialdehyde and higher levels of GPX4 compared to controls. Similarly, metformin protected THP-1 cells from erastin-induced ferroptosis by lowering HMOX1 levels. This suggests that lowering HMOX1 protects THP-1 cells from ferroptotic cell death (135). Additionally, erythrophagocytosis-induced ferroptosis in phagocytes exacerbates plaque progression in late-stage atherosclerosis. Decreasing intraplaque ferritin heavy chain and HMOX1 expression via treatment with a pharmacological ferroptosis inhibitor (UAMC-3203) slowed progression in atherosclerotic plaques with intralesional hemorrhage (136). Ferroptotic cell death was also implicated in worse outcomes in a model of acute myocardial infarction, and both treatment with an iron chelator or genetic deletion of BACH1 extended survival and decreased infarct size, in part through upregulation of the heme oxygenase system and glutathione synthesis enzymes (16).

Taken together, this literature suggests a complicated role for heme oxygenase in the myeloid niche in varied disease settings. This can likely be attributed to the redox state of the tissue in each pathology and the importance of maintaining a cellular redox balance that prevents lipid oxidation and ultimately, ferroptotic cell death.

## Conclusions

**Figure 2:**
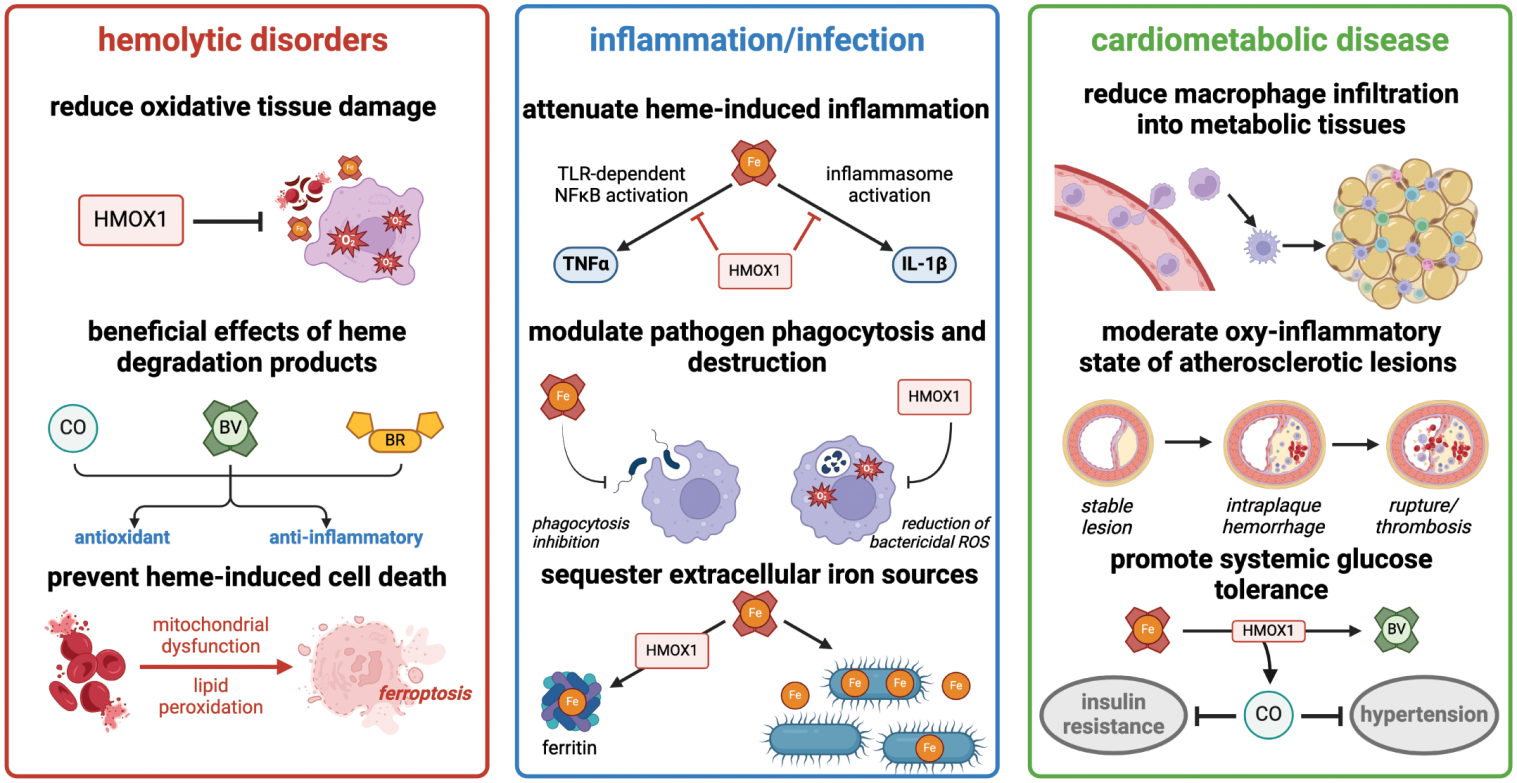
Heme degradation by HMOX1 has diverse effects in hemolytic disorders, acute inflammation, and cardiometabolic disease. Summary of major consequences of heme detoxification in these clinically-relevant processes. Figure created with BioRender (http://biorender.com).

The pathological role for heme in hemolysis, inflammatory disorders, and cardiovascular disease has become an increasingly important area of investigation in the pre-clinical and translational realm (Summarized in **Figure 2**). Macrophages are the key regulators of heme homeostasis, as they utilize the heme oxygenase system to minimize the oxidative and inflammatory effects of free heme. Mechanistic studies of HMOX1 function in macrophages have not only revealed the impact of heme clearance on acute and chronic inflammation and oxidative stress, but also uncovered a nuanced, context-dependent role for HMOX1 in either attenuating or promoting inflammation and ferroptosis. We particularly want to acknowledge the many excellent studies that have contributed to this field but are not included in the present review due to space and content limits. As a result of this continually-evolving body of work, pharmacological activation or inhibition of HMOX1 activity, as well as administration of heme breakdown products, including CO and bilirubin, have emerged as exciting therapeutic targets for treatment of a diverse array of disorders. Future work in the field will undoubtedly refine our understanding of the balance between heme detoxification, oxidative stress, and inflammation and provide translationally-relevant insights into the treatment of heme-driven diseases.

## Author contributions

SY: Conceptualization, Visualization, Writing – original draft, Writing – review & editing. CU: Writing – original draft, Writing – review & editing. NL: Conceptualization, Writing – original draft, Writing – review & editing.
